# Filopodial-Tension Model of Convergent-Extension of Tissues

**DOI:** 10.1371/journal.pcbi.1004952

**Published:** 2016-06-20

**Authors:** Julio M. Belmonte, Maciej H. Swat, James A. Glazier

**Affiliations:** 1 Biocomplexity Institute and Department of Physics, Indiana University Bloomington, Bloomington, Indiana, United States of America; 2 Biocomplexity Institute and Department of Intelligent Systems Engineering, Indiana University Bloomington, Bloomington, Indiana, United States of America; Utrecht University, UNITED STATES

## Abstract

In convergent-extension (*CE*), a planar-polarized epithelial tissue elongates (*extends*) in-plane in one direction while shortening (*converging*) in the perpendicular in-plane direction, with the cells both elongating and intercalating along the converging axis. CE occurs during the development of most multicellular organisms. Current CE models assume cell or tissue asymmetry, but neglect the preferential filopodial activity along the convergent axis observed in many tissues. We propose a cell-based CE model based on asymmetric filopodial tension forces between cells and investigate how **cell-level** filopodial interactions drive **tissue-level** CE. The final tissue geometry depends on the balance between external rounding forces and cell-intercalation traction. Filopodial-tension CE is robust to relatively high levels of planar cell polarity misalignment and to the presence of non-active cells. Addition of a simple mechanical feedback between cells fully rescues and even improves CE of tissues with high levels of polarity misalignments. Our model extends easily to three dimensions, with either one converging and two extending axes, or two converging and one extending axes, producing distinct tissue morphologies, as observed *in vivo*.

## Author Summary

The development of an embryo from a fertilized egg to an adult organism requires not only cell proliferation and differentiation, but also numerous types of tissue restructuring. The development of a relatively round initial embryo into one elongated along its rostral-caudal axis involves coordinated tissue elongation and cell reorganization in one or more groups of cells or tissues. Counterintuitively, in many organisms, cells in elongating tissues elongate and increase their protrusive activity in the direction **perpendicular** to the axis of elongation (*convergent extension*). Experimental and theoretical studies have not determined how this **cell-level** oriented protrusive activity leads to observed **tissue-level** changes in morphology. We propose a filopodial-tension model that shows how tension from oriented cell protrusions leads to observed patterns of tissue CE.

## Introduction

**Fig 1:**
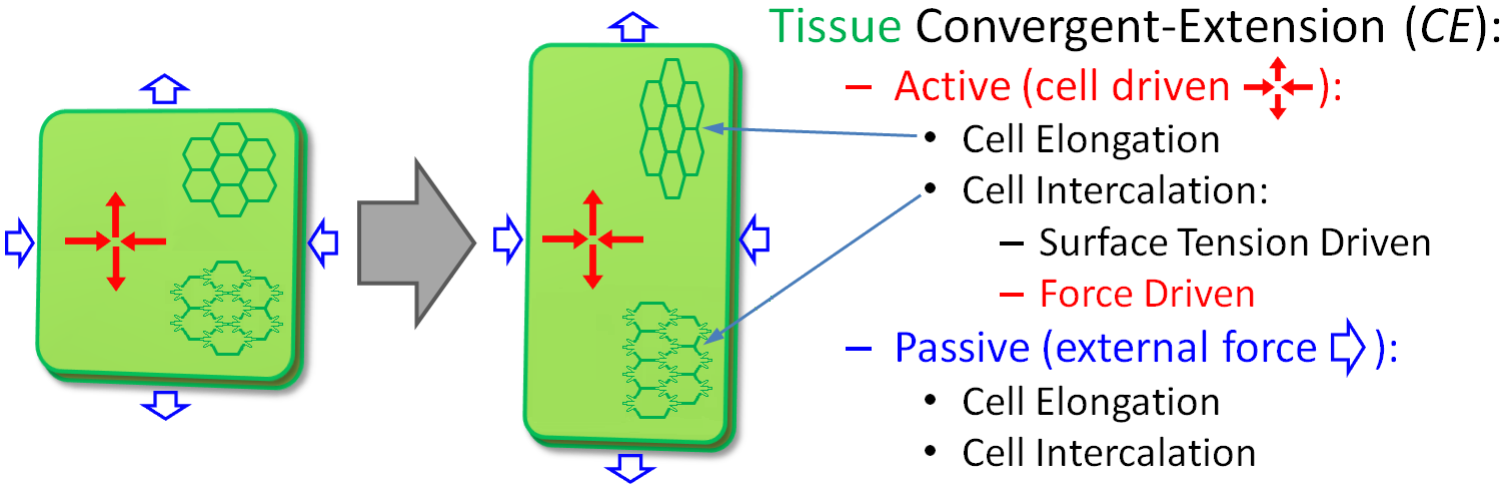
Types of convergent extension. In *active* convergent-extension, the cells in the tissue generate deforming forces due to anisotropic adhesion or pulling forces between cells (red arrows), while in *passive* convergent-extension, the surrounding environment deforms the tissue (blue arrows). Cell intercalation occurs in types of CE, but the axis of cell elongation is typically perpendicular to the axis of elongation in active CE and parallel in passive CE.

Embryonic development requires numerous changes in tissue morphology. *Convergent-extension* (CE) is a basic tissue shape change [1–9], during which cells in an epithelial sheet rearrange to narrow (*converge*) the tissue along one planar axis while lengthening (*extending*) it along the perpendicular planar axis (Fig 1). Although CE has been observed in the development of many organisms [1–8], the specific cellular mechanisms that drive such movements are still subject of investigation [10].

Both asymmetric external forces on a tissue (*passive CE*) and asymmetric forces generated by the cells within a tissue (*active CE*) can lead to CE (Fig 1) [10]. Hypothesized mechanisms for CE include anisotropic cell edge/actin contraction [11,12], anisotropic cell adhesion and elongation [13,14], cell shape extension/retraction [11,15], combinations of a constraining boundary with undirected cell elongation [16] or with directed leading edge protrusion [17], and increased cell adhesion within tissue segments [18] (see Supplemental Material for a more detailed discussion of previous models). Existing models of CE, however, neglect the experimentally observed prevalence of filopodial extension parallel to the direction of tissue convergence [3,9,19–24], which could produce anisotropic traction forces between cells or between cells and the extracellular matrix [25–28].

The observed asymmetry of filopodial protrusion led us to propose a filopodial-tension mechanism for CE based on anisotropic filopodial pulling forces between cells. We explicitly model the number of cell-cell connections, their range, angular distribution, strength, and frequency of formation and breakage. We define an appropriate set of metrics to quantify both the effects of model parameters and planar-polarization defects (such as misalignments and the passive cells) on the dynamics of tissue-level CE. Since our filopodial-tension model extends naturally to three dimensional tissues, we discuss the two types of 3D CE and their corresponding tissue morphologies.

## Methods

### Anisotropic Filopodial-Tension Model of Convergent Extension

Experiments show that long filopodia continuously form and retract during CE in epithelial sheets and that these filopodia preferentially form in-plane along angles near the axis of tissue contraction. Each model cells therefore extends and retracts filopodia (which we represent using the model concept of a *link*) distributed within a range of angles around the directions perpendicular to the cell’s planar-polarity axis. To simulate the observed binding of filopodial tips to other cells and the roughly length-independent pulling forces which retracting filopodia generate, in our model, an extending link binds to the cell it contacts, then generates a constant (length independent) tension force between the cells it connects [20,25,29]. We then test whether this tension force is sufficient to explain observed local cell intercalation and global tissue CE.

**Fig 2:**
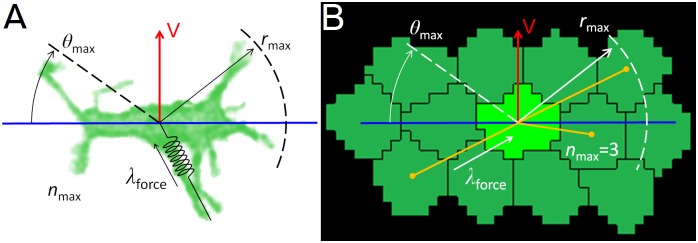
Cell intercalation model. (**A**,**B**) Given a planar-polarization vector (red) and convergence axis (blue), a **cell** forms links with up to n_max_ **cells** that lies within the interaction range r_max_ from its center-of-mass and within an angle ±ϑ_max_, of the convergence axis. Each link exerts a tensions force λ_force_ on both of the **cells** it connects. (**A**) Image of a bipolar cell in chicken limb-bud mesenchyme overlaid with model parameters. (**B**) Snapshot of a GGH/CPM computer simulation of the filopodial-tension model, overlaid with model parameters. Dark yellow lines represent simulated filopodial links between a **cell** (light green) and its currently interacting neighbors (dark green). Experimental image courtesy of Gaja Lesnicar-Pucko and James Sharpe, CRG, Barcelona.

In the filopodial-tension model (Fig 2, S1 Movie) **cells** form and eliminate **links** representing filopodia with a defined set of neighboring **cells** (terms in **boldface** identify model objects). Each **cell** carries a polarization vector (perpendicular to its planar-polarity axis) (Fig 2, red arrow) that defines its preferred direction of filopodial protrusion (Fig 2, blue horizontal line). We simplify the model by having the links connect the centers-of-mass of **cells** rather than connecting the actin cortex of one cell to the actin cortex of the contacted cell, as do real filopodia. Because filopodia typically form in a pair of growth cones roughly along the convergence axis and with a typical maximal length, we allow a **cell** to form links within a range of angles ±*ϑ*_max_ around this axis on either side of the **cell** with a maximum length of approximately *r*_max_. Specifically, a **cell** can form a link only with those **cells** whose centers-of-mass lie within a distance *r*_max_ from its center of mass and within an angle ±*ϑ*_max_ of its polarization axis (Fig 2, blue horizontal line). A **cell** can have at most *n*_max_ links to other **cells** at any time (including links formed and received) and only one link is allowed between any pair of **cells**. The actual number of links a **cell** forms may be less than *n*_max_. Each link between a pair of **cells** exerts a tension force of magnitude *λ*_force_ along the line connecting the **cells’** centers-of-mass. To model the finite lifetimes of filopodia, we define a relaxation time, *t*_interval_, after which we remove the links of all **cells** and create new ones. In a simulation in which the links form and then persist indefinitely, the **cells** only move a few microns (lattice sites) from their original locations and the **tissue** does not converge or extend.

We implement the filopodial tension model using the Cellular Potts model (*CPM*, also known as the Glazier-Graner Hogeweg model, *GGH*), where each cell is represented as a collection of lattice sites with the same **cell** index. An effective-energy cost function, *H*, specifies the **cell’s** properties (see supplemental material). The tension force along a link between a pair of **cells** is independent of its length and acts along the vector between their centers-of-mass. In the GGH/CPM formalism, the tension has the form:

$$H=H_{0}+\sum_{\sigma ,\sigma \prime }\lambda_{\text{force}}\left(\sigma ,\sigma \prime \right)l_{\sigma ,\sigma \prime }$$ (1)

where the sum is over all pairs of linked **cells**, *λ*_force_ is the strength of the pulling force between **cells** *σ* and *σ’*, *l*_*σ*,*σ’*_ is the current distance between the **cells**, and the term *H*_*0*_ aggregates all the other GGH/CPM cost function terms. The GGH/CPM simulations evolve stochastically from random lattice-site updates subjected to the effective-energy cost function, *H*. The time unit is the *Monte Carlo Step* (*MCS*), defined as the rate of lattice-site updates (see supplemental material for more details on the GGH/CPM formalism).

The filopodial-tension model has five intensive parameters (*λ*_force_, *t*_interval_, *r*_max_, *n*_max_, *ϑ*_max_) and one extensive parameter (*N*, the number of **cells**), making a complete sensitivity analysis computationally costly. We therefore fixed all parameters to reference values that are within the ranges observed *in vivo* and produced biological plausible convergent-extension (Table 1), then studied the effects of varying each intensive parameter one-at-a-time. The biological parameters proposed by the model can be directly measured experimentally, but since the concept of a filopodial-based CE is new and applies more readily to CE of deep tissues, which are not as easily visualized as epithelial sheets, appropriate experimentally-derived values are harder to find. The most studied cases are chicken limb-bud mesenchymal intercalation [30] (*t*_interval_ = 2.2 hours; *r*_max_ = 3 cell diameters; *n*_max_ = 11; *ϑ*_max_ = 45°), *Xenopus* gastrulation and notochord formation [31–33] (*t*_interval_ = 2.0–2.7 min; *r*_max_ = 1.5 cell diameters; *n*_max_ = 8–9; *ϑ*_max_ = 60°), and *Xenopus* Keller explants [23,34] (*t*_interval_ = 0.5–1.0 hour; *r*_max_ = 1.5 cell diameters; *n*_max_ = 8–9; *ϑ*_max_ = 30°).

**Table 1:** List of reference parameters values used in the simulations. Parameter sweeps vary one of the first 7 parameters, while keeping all the others constant. Key: λ_force_, pulling strength; t_interval_, time interval between link formation/breakage (MCS); r_max_, maximum distance between **cells** (**cell** diameters); n_max_, maximum number of links per **cell**; ϑ_max_, maximum angle (radians); N, number of **cells**; cd, **cell** diameter (lattice sites); T, temperature or level of noise in the simulations; λ_volume_, **cell** stiffness; n_orders_, neighboring orders for lattice site flip and contact energy; J_c,M_, adhesion energy between **cells** and **medium**; J_c,c_, adhesion energy between **cells**.

Type of parameter	Filopodial tension model					Spatial	GGH/Cellular Potts model					
Parameter	*λ*_force_	*t*_interval_	*r*_max_	*n*_max_	*ϑ*_max_	*N*	*cd*	*T*	*λ*_volume_	*n*_orders_	*J*_c,M_	*J*_c,c_
**2D**	50	20	2 cd	3	π/4	109	10	50	5	2, 4	10	10
**3D**	500	50	2 cd	3	π/4	552	6	80	5	2, 5	10	10

### Metrics

**Fig 3:**
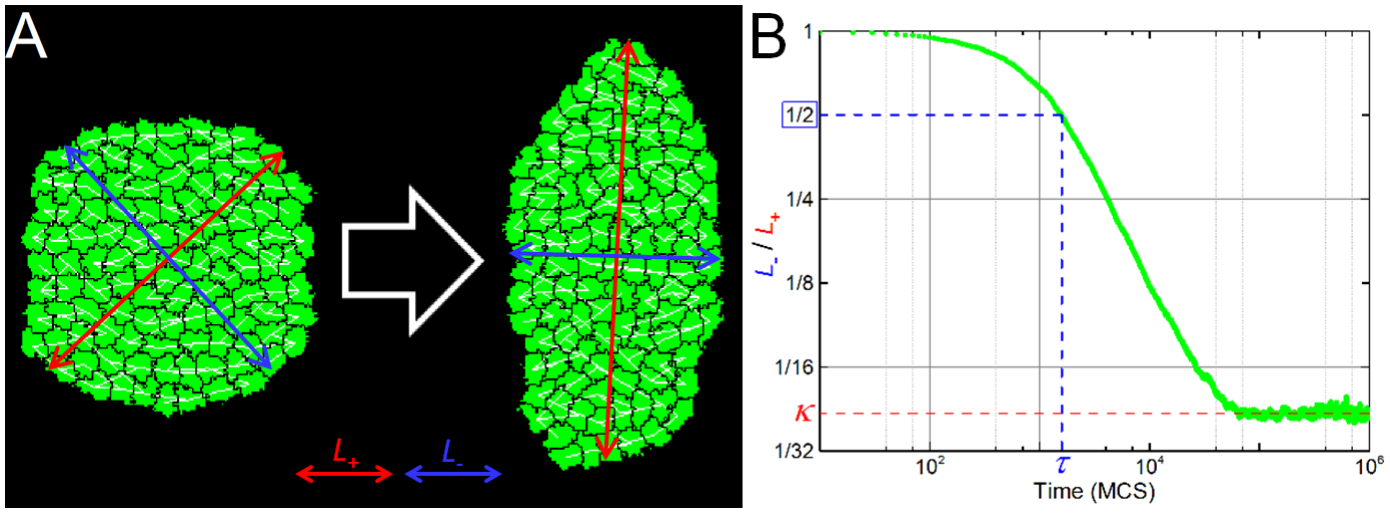
Simulation snapshots and metrics. (**A**) Snapshots of a 2D simulation with reference parameter values, showing the initial configuration (left) and the configuration when the length of the major axis (L_+_, red lines) increases to twice the length of the minor axis (L_-_, blue lines), i.e., κ = 0.5. The simulation contains N = 109 **cells** (in green) with the tension forces shown by the white segments connecting their centers-of-mass. (**B**) Graph of L_-_/L_+_ versus time for the reference 2D simulation. For all simulations we measured the final value of the ratio between the length of minor and major axes of the tissue κ (shown in red), and the time τ (shown in blue) when the length of the major axis doubles the length of the minor axis (L_-_/L_+_ = 0.5).

All simulations start with a mass of identical **cells** uniformly distributed inside a rough circle. Each **cell** has the same planar-polarization vector (**V**). To quantify the degree of **tissue** deformation we calculate the *inverse aspect ratio* between the length of the minor (*L*_*-*_) and major (*L*_*+*_) axes of the **tissue** (Fig 3B, green line). Initially the aspect ratio is close to 1 and decreases in time to a final value *κ* (Fig 3B, dashed red line) that depends on the **filopodial** tension parameters (*λ*_force_, *t*_interval_, *r*_max_, *n*_max_, *ϑ*_max_), the number of **cells** in the tissue (*N)* and the surface tension of the **tissue** *γ* (defined below).

The final inverse aspect ratio quantifies the maximum elongation of the **tissue**, but does not convey how fast the **tissue** elongates. To quantify the elongation rate, we define the *elongation time* (*τ*) the time an initially isotropic **tissue** takes for its major axis (*L*_+_) to double the length of its minor axis (*L*_-_), which is equivalent to the time when the inverse aspect ratio (*L*_*-*_/*L*_*+*_) first decreases to 0.5 (Fig 3B, dashed blue lines). We consider CE to fail if *L*_*-*_/*L*_*+*_ never reaches 0.5.

Since both the filopodial-tension model and the GGH/CPM are stochastic, we average the value of the elongation time (*τ*) over 10 simulation replicas. Because the **tissue** inverse elongation ratio converges to the same value independent of the simulation seed or initial conditions (S1 Fig), unless specified otherwise, we calculate the final inverse aspect ratio *κ* for a single simulation replica, with the standard deviation indicating the fluctuations in *κ* around its final value for that replica.

## Results

### Surface Tension vs. Filopodia Tension

Successful CE depends on the ability of intercalating cells to generate forces stronger than the internal and external forces that oppose tissue deformation. Here, the opposing forces come from the superficial tension (*γ*) between the **cells** and the external **medium**, defined as [35]:

$$\gamma =J_{\text{c,M}}-\frac{J_{\text{c,c}}}{2},$$ (2)

where *J*_c,c_ is the contact energy between **cells** and *J*_c,M_ is the contact energy between **cells** and **medium** (see supplemental material).

**Fig 4:**
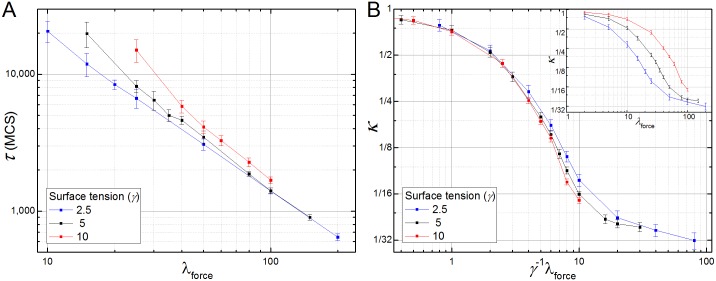
Competition between filopodial tension and surface tension in the 2D filopodial tension model. (**A**) The elongation time (τ) till the **tissue’s** inverse aspect ratio decreases to 0.5 as a function of the filopodial tension (λ_force_) of the cells for different surface tensions (γ). (**B**) Insert: Degree of tissue deformation (κ) as a function of λ_force_. An increase in the surface tension of the tissue reduces the final degree of CE (larger κ) shifting the κ vs. λ_force_ curve to the right. The opposite effect happens when the surface tension is decreased. Main: The κ vs. λ_force_ curves collapse when we rescale with the tension force by the surface tension plotting κ vs. λ_force_/γ.

When the filopodial tension is weak compared to the surface tension (*λ*_force_ *<* 2*γ*), **cells** do not intercalate and CE fails. For larger filopodial tensions, the elongation time (*τ*) decreases as a power of *λ*_force_ (*τ* ∝ *λ*_force_^-1.25±0.03^) (Fig 4A, red line). The final inverse aspect ratio (*κ*) decreases monotonically with increasing *λ*_force_ (Fig 4B). Increasing *γ* shifts the *κ* vs. *λ*_force_ curve to the right and decreasing *γ* shifts the *κ* vs. *λ*_force_ curve to the left (Fig 4B, inset). Normalizing the filopodial tension by the surface tension (*λ*_force_/*γ*) collapses the *κ* vs. *λ*_force_ curves (Fig 4B), showing the linear relationship between *λ*_force_ and *γ*. The surface tension (*γ*), however, has little effect on the elongation time (*τ*), which depends on *λ*_force_, but is relatively insensitive to surface tension (Fig 4A). The *κ* vs. *λ*_force_/*γ* curve is sigmoidal on a log-log scale (Fig 4B), because the shape of the **tissue** changes little for weak filopodial tensions and because the total number of **cells** limits *κ* for strong filopodial tensions (see Fig S3A). At the inflection point of *κ* vs. *λ*_force_/*γ*, the tensions of the links (*λ*_force_) balances the external surface-tension forces that oppose **tissue** elongation (*λ*_force_/*γ* ~ 6). Near this inflection point *κ* varies as an approximate power law of *λ*_force_ (*κ* ∝ *λ*_force_^-1.51±0.08^).

### Parameter Sensitivity

Next we studied how the remaining filopodial tension parameters affect CE, specifically, the mean lifetime of the filopodia, modeled as the time interval between **link** formation and breakage (*t*_interval_); the maximum length of the filopodia, modeled as the maximum distance of interaction between the **cells**’ centers-of-mass (*r*_max_); the maximum number of filopodial interactions per **cell** (*n*_max_); and the maximum angle between the filopodial direction and the **cells**’ convergence axis (*ϑ*_max_).

**Fig 5:**
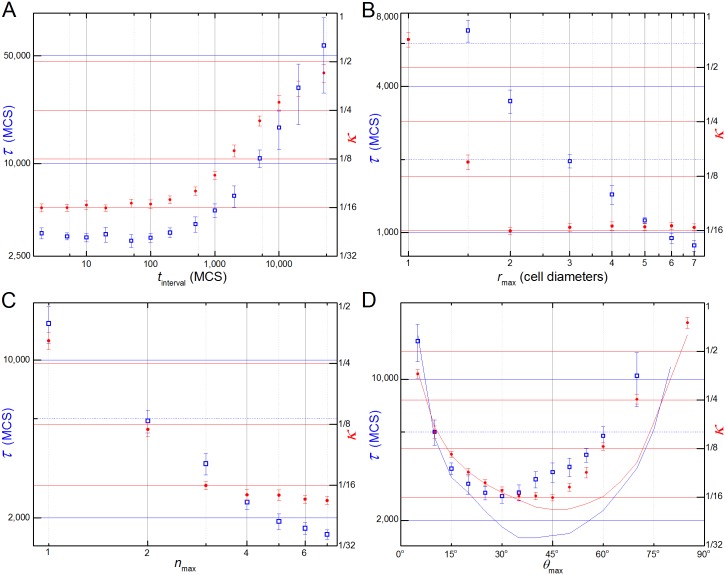
Parameter sensitivities. Left vertical axes and open blue squares correspond to τ and right vertical axes and solid red dots corresponds to κ. Parameters changes one-at-a-time with remaining parameter set to their reference values. (**A**) Filopodial lifetime (t_interval_): κ and τ are independent of t_interval_ for lifetimes lower than the typical time for **cell** rearrangement (t_interval_ < 200 MCS) and increase monotonically for t_interval_ > 200 MCS. (**B**) Filopodial range (r_max_): κ decreases with increasing r_max_ for r_max_ < 2 **cell** diameters and is constant for r_max_ > 2 **cell** diameters. τ decreases monotonically with increasing r_max_. CE fails for r_max_ < 1.5 **cell** diameters. (**C**) Number of filopodial interactions (n_max_): κ and τ decrease monotonically with increasing n_max_, however κ decreases more slowly for n_max_ > 4. (**D**) Angular range of **filopodia** (ϑ_max_): for n_max_ = 3 (reference value), τ (blue squares) and κ (red dots) decrease monotonically with increasing ϑ_max_ for small ϑ and increase monotonically with increasing ϑ_max_ for large ϑ, with minima at ϑ_max_ = 30° and ϑ_max_ = 40°, respectively. CE fails for ϑ_max_ > 70°. For n_max_ = 7, τ vs. ϑ_max_ (blue line) and κ vs. ϑ_max_ (red line) are also concave curves with minima at ϑ_max_ = 40° and ϑ_max_ = 50°, respectively. CE fails for ϑ_max_ > 80°.

Fig 5A shows that, for the reference parameter values (Table 1) the lifetime of **filopodia**, *t*_interval_, has no effect on *τ* or *κ* for *t*_interval_ ≲ 200 MCS. For the reference parameter values, 200 MCS corresponds to the typical time the **cells** require to rearrange their positions in response to a given set of **neighbors** interactions. Increasing filopodial lifetimes above 200 MCS slows **cell** intercalation (increasing the elongation time) and increases the **tissue’s** final inverse aspect ratio (corresponding to less deformation).

The maximum range (*r*_max_) of filopodia interaction has different effects on the final inverse aspect ratio (*κ*) and elongation time (*τ*). For *r*_max_ < 2 **cell** diameters, *κ* decreases as a power law in *r*_max_ (*κ* ∝ *r*_max_^-3.5±0.2^), then saturates for *r*_max_ ≥ 2 **cell** diameters, while the elongation time (*τ*) decreases monotonically with increasing *r*_max_ (Fig 5B). The same effect is seen with respect to the maximum number of links (*n*_max_): *κ* decreases as a power law in *n*_max_ for *n*_max_ < 4 (*κ* ∝ *n*_max_^-1.5±0.03^) and saturates for *n*_max_ > 4 (this saturation makes sense since the **cell** typically has 4 **neighbors** within the range of its filopodia for *r*_max_ = 2 and *ϑ*_max_ = 45°) while *τ* decreases monotonically with increasing *n*_max_ (Fig 5C). Thus *r*_max_ and *n*_max_ have affect the rate of **cell** intercalation more than the final inverse aspect ratio, while the **tissue’s** surface tension affects only the final inverse aspect ratio and not the rate of **cell** intercalation (Fig 4).

Both *κ* and *τ* are concave with respect to the maximum angle of filopodial protrusion (*ϑ*_max_), since for small *ϑ*_max_ the number of **cell** center-of-mass within the cones defined by *ϑ*_max_ and *r*_max_ is very small, while for *ϑ*_max_ = 90° the forces on the **cell** are symmetric since it extends **filopodia** uniformly in all directions. In both limits CE fails (Fig 5D). Since the net intercalation force is the difference between the tension forces parallel and perpendicular to the convergence axis (roughly $\int_{0}^{\theta_{max}}(\text{cos}\left(\theta \right)-\text{sin}\left(\theta )\right)d\theta \;$), we might expect the force to be greatest (and thus *κ* and *τ* to be smallest) when *ϑ*_max_ = 45° and for their values to increase symmetrically away from *ϑ*_max_ = 45°. The curves, however, have different minima and are not symmetric: the smallest final inverse aspect ratio (*κ*) is around *ϑ*_max_ *=* 40° (Fig 5D, red dots) and the smallest elongation time (*τ*) is around *ϑ*_max_ *=* 30° (Fig 5D, blue squares).

This asymmetry is caused by the limited number of **neighbors** with which a **cell** can form a link. Both the maximum number of links per **cell** (*n*_max_) and the number of **cells** within the link interaction range (*r*_max_) can limit the actual number of links a **cell** forms. If the maximum number of links per **cell** is lower than the number of **cell** neighbors within a cone of range *r*_max_ (e.g. *n*_max_ = 3) and angle *ϑ* < *ϑ*_max_, increasing *ϑ*_max_ leads to more links with **cells** at larger *ϑ* and thus reduces the net tension force applied along the direction of the convergence axis. In effect, large *ϑ*_max_ causes the **cell** to waste its limited number of **filopodia**. For large *n*_max_, **links** form to all **cells** within the cone of range *r*_max_ and small *θ* regardless of the value of *ϑ*_max_.Thus, for large *n*_max_ (e.g. *n*_max_ = 7), the *κ* and *τ* curves are roughly symmetrical around their minima at *ϑ*_max_ *~* 45° (blue and red lines in Fig 5D).

### Contact-Mediated Pulling

**Fig 6:**
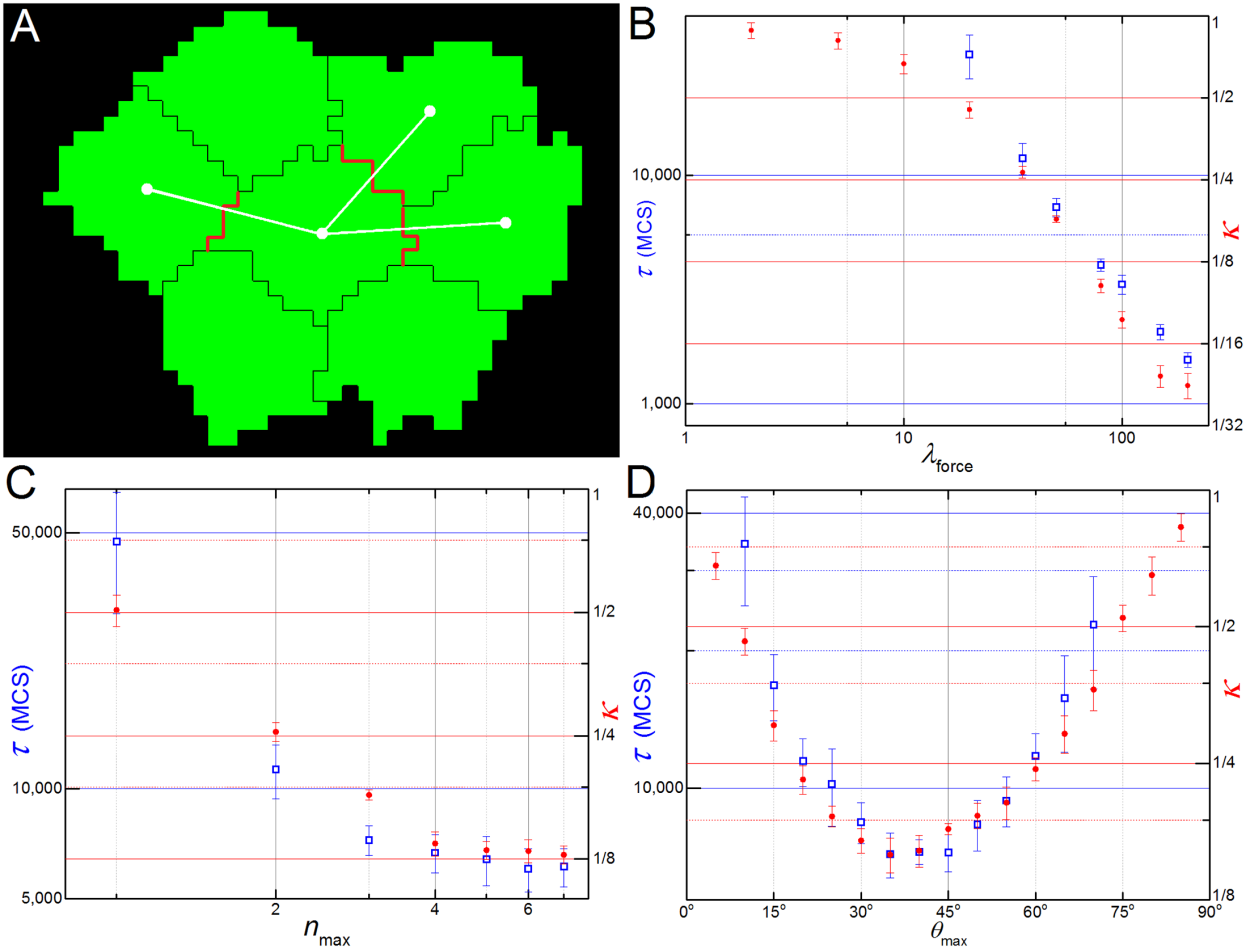
Contact-mediated pulling version of the model. (**A**) **Cells** only pulls **neighbors** (here, 3) that share a common surface area (shown in red) and that lie inside a maximum angle with respect to the convergence plane (here the horizontal axis). (**B**) Dependence of τ and κ with λ_force_ is qualitatively the same as before (Fig 4). (**C**) Dependence with n_max_ is reversed, with the speed of intercalation (τ^-1^) saturating after n_max_ = 3 and κ still decreasing. (**D**) The (κ τ,) x ϑ_max_ curves are more symmetric, but the **tissue** still elongates more and faster at lower angles.

The filopodial tension model assumes that cells can extend filopodia, contact and pull other cells that lie within a given distance, even if they do not touch each other before filopodial extension. An example would be the formation of adhesion junctions between cells which coupled to a contractile stress fiber in both cells. To model these cases, we defined a *contact-mediated cell tension model*, which is identical to the filopodial tension model except that the maximum link length *r*_max_ in the filopodial tension model is replaced with the condition that **cells** must be in touch before pulling on each other (Fig 6A).

The qualitative results for the contact-mediated cell tension model do not differ much from the filopodial tension model. The *κ* x *λ*_force_ curve is sigmoidal on a log scale, *τ* decreases with a power law (*κ* ∝ *λ*_force_^-1.18±0.06^) and CE fails for *λ*_force_ < 20 (Fig 6B). The dependence of *κ* on the number of filopodial interactions (*n*_max_) is still a power law (*κ* ∝ *n*_max_^-1.5±0.03^) and saturates when *n*_max_ = 4. The elongation time (*τ*), however, does not keep decreasing as it does for the filopodial tension model, but also saturates around *n*_max_ = *4* links (Fig 6C), as few **cells** have more than 4 **neighbors** with centers near the convergence plane. The (*κ*, *τ*) x *ϑ*_max_ curves have minima at *ϑ*_max_ *=* 40° and *ϑ*_max_ *=* 35°, respectively, but are less skewed than in the filopodial tension model (compare Figs 6D and 5D). CE fails for *ϑ*_max_ *<* 10° and *ϑ*_max_ *>* 70°.

### Polarization Misalignment

Convergent-extension requires cells to have consistent planar polarity throughout an extensive region of tissue. This correlated orientation might result from a long-range bias from a morphogen gradient, cellular or intercellular differences in protein expression [36], or from a boundary-relay mechanism [37,38]. In our previous simulations we assumed that all **cells** had perfectly aligned polarization vectors (Fig 2, red arrows), *i*.*e*., they all pointed in the same direction with the same magnitude, and they maintained their internal orientation throughout the simulation. To study the effect of polarization misalignment on CE we added a zero-mean Gaussian distributed displacement angle to the **cells’** polarization vectors and varied the standard deviation of the distribution (*σ*) while keeping the mean direction (here, the vertical axis) constant. Since the final elongation ratio is sensitive to the distribution of polarization vectors, the values of *κ* were averaged over 5 simulations.

**Fig 7:**
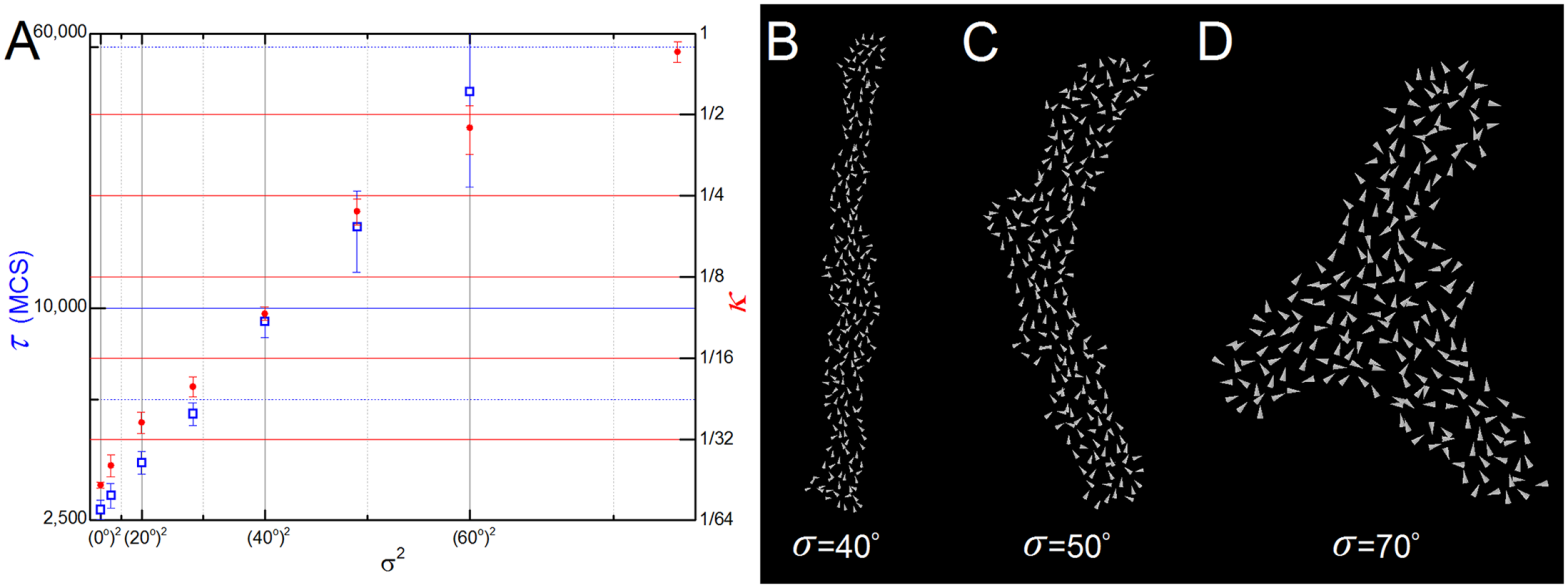
Simulation results for different levels of polarization misalignment. (**A**) Semi-log graph of τ and κ with the variance (σ^2^). Both metrics are exponential functions of the variance. (**B-D**) Snapshots of 3 simulations with different levels of misalignment (σ = 40°, 50° and 70°). Each cell is represented by a white vector showing the direction of its polarization. The bigger vectors on (**C**) and (**D**) are due to zoom.

The filopodial tension model tolerates small polarization misalignments, with a **tissue** with a displacement angle of *σ =* 10° reaching the same final inverse aspect ratio as in the perfectly aligned case with little decrease in elongation rate (an 11% increase in *τ*). The **tissue** remained aligned with the mean direction of **cell** polarization (the vertical axis) for small misalignments (*σ <* 40°, Fig 7B), but bent at around *σ =* 50° (Fig 7C). For polarization misalignments with *σ >* 60°, CE fails and the **tissue** breaks its symmetry, acquiring more complex shapes such as the caltrop (see Fig 7D). Both metrics are exponential functions of the variance *σ*^2^ (Fig 7A).

### Mechanical Feedback Rescue of CE

So far we have assumed that the polarization vector of the cells remains constant throughout the entire process. In reality, however, cells are constantly communicating with their neighbors either through signaling or through mechanical interactions. During CE cells establish and maintain their polarity through the planar cell polarity (PCP) pathway, however there is growing evidence that mechanical feedback may also play a role in the maintenance of global tissue polarity during development [39–42].

**Fig 8:**
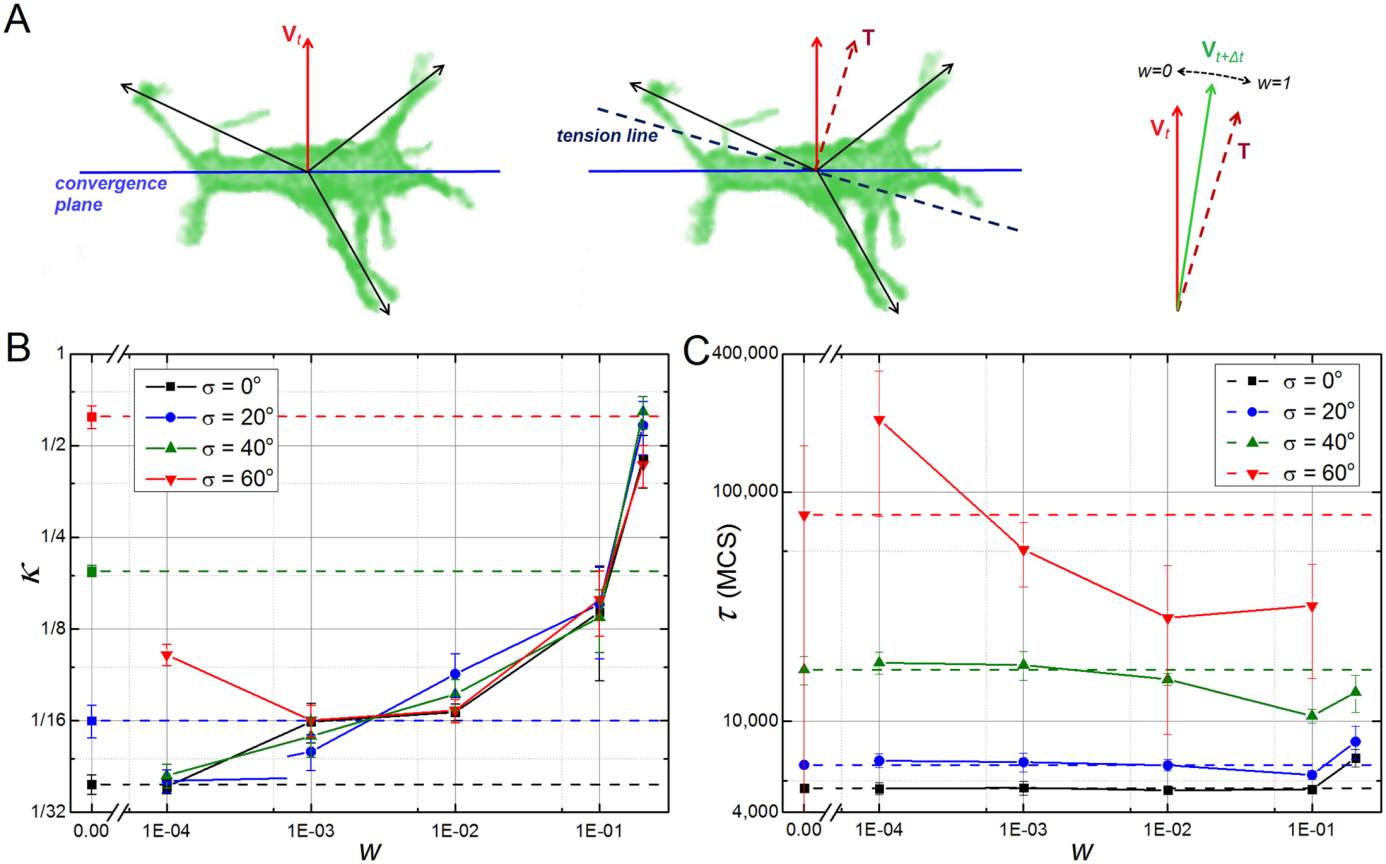
Mechanical feedback rescues CE on tissues with polarization misalignment. (**A**) Schematic view of the mechanical feedback model: every **cell** has a polarization vector V_t_ (red solid arrow) that defines an orthogonal convergence plane (blue solid line); at time t the **cell** pulls and it is pulled by it neighbors; this set of pulling forces (black arrows) defines a tension line along the cell (dashed dark blue line) which in turn defines an orthogonal vector T; at time t+Δt a new polarization vector V_t+Δt_ is set by the weighted average of T and V_t_, where w is the feedback factor with w = 0 corresponding to the case with no feedback and w = 1 corresponding to no memory of previous orientations. (**B**) Dependency of parameter κ with the feedback weighting factor w in **tissues** with different levels of tissue misalignment. Horizontal dashed lines indicate results with no feedback (w = 0). High feedback worsens final elongation for **tissues** with low polarization misalignment (w ≥ 0.001 for σ = 0° and w ≥ 0.01 for σ = 20°), but in general rescues and even leads to higher elongation ratios with weaker feedback levels. (**C**) Dependency of parameter τ with the feedback weighting factor w in **tissues** with different levels of tissue misalignment. Horizontal dashed lines indicate results with no feedback (w = 0).

The presence of a mechanical feedback mechanism may rescue CE in tissues with high polarization alignment defects. In order to investigate this we developed a simple model of mechanical feedback and applied it to the misalignment polarization cases that have been described in the last section (Fig 7). The feedback model assumes that the pulling forces on a **cell** due to **filopodial** interactions affect its polarization vector. We implement a simple phenomenological version of such an interaction by calculating the line of tension from the sum of all filopodial interactions of the **cell** with its **neighbors** (Fig 8A). From this line of tension we extract an orthogonal vector ***T*** which is averaged with the previous cell polarization vector ***V*** in the following way:

$$V_{\text{t+}\Delta \text{t}}=V_{\text{t}}^{*}\left(1-w\right)+T_{\text{t}}^{*}w,$$ (3)

where ***V***_t+Δt_ is the polarization vector of a **cell** at time *t+Δt*, ***V***_t_* is the normalized polarization of the same **cell** at time *t*, ***T***_t_* is the normalized tension vector of the **cell** at time *t*, and *w* is a feedback weighting factor ranging from 0 (no feedback) to 1 (no memory) (Fig 8A). This iterative processes repeated at discrete time intervals set equal to filopodia lifetime (Δt = *t*_interval_). For simplicity, we do not distinguish between the pulling forces generated by the **cell** from the pulling forces that their neighbors exert on it. The normalized tension vector of a **cell** is calculated from the vector that maximizes the sum of all projections of the normalized lines of force from all the **cells**’ neighbors:

$$\Sigma_{i}\;\text{cos}(\emptyset_{\text{i}}-\;\emptyset_{\text{T}})=\;0,$$ (4)

where the sum is over all the **cell**’s neighbors that pull on it, *ϕ*_i_ is the angle of the line of force between the **cell** and the pulling neighbor *i*, and *ϕ*_**T**_ is the angle that defines the tension vector T* *= (*cos *ϕ*_**T**_, sin *ϕ*_**T**_*)*.

For **tissues** with a high starting level of polarization misalignment (*σ ≥* 40°), addition of this mechanical feedback mechanism usually leads to a lower final elongation ratio *κ*, as long as the feedback factor *w* is below 0.1. For these cases, **tissue** elongation times (*τ*) decrease with *higher* feedback levels (Fig 8C, green and red lines), while *k* usually decreases with *lower* feedback levels. For **tissues** with a low starting level of polarization misalignment (*σ ≤* 20°), addition of this mechanism leads to lower final elongation ratios only for small levels of feedback (*w ≤* 0.001) (Fig 8B, blue and black lines), while the time of tissue elongation (*τ*) remains relatively unchanged with respect to the case with no feedback (Fig 8C, blue and black lines). In all simulations where the addition of mechanical feedback rescues CE, the **cells** established a global polarization axis emergently. We chose to implement the feedback update iteratively, which leads to fast destabilization of the **tissue** for high levels of feedback, as is expected for a case with no memory. Although a continuous model would be relatively more robust, we expect the same destabilization effect when the weighting factor *w* approaches 1.

### CE in the Presence of Non-active Cells

Next we varied the number of intercalating **cells** in the **tissue** to check if there is a minimum number of active **cells** needed to drive CE and how this change **tissue** dynamics. We defined two types of **cells** without filopodia: *passive* **cells**, which lack **filopodia** but can be pulled by the **filopodia** of other **cells**; and non-responsive, or *refractory* **cells**, which cannot be pulled by the **filopodia** of other **cells**. The former would correspond to cells whose surface adhesion molecules were compatible with those of the cells extending filopodia and the latter to cells with incompatible adhesion molecules. The parameters for **cells** which produced **filopodia** were the same as in Table 1. Since the final elongation ratio is sensitive to the distribution of active/non-active **cells**, the values of *κ* were averaged over 5 simulations.

**Fig 9:**
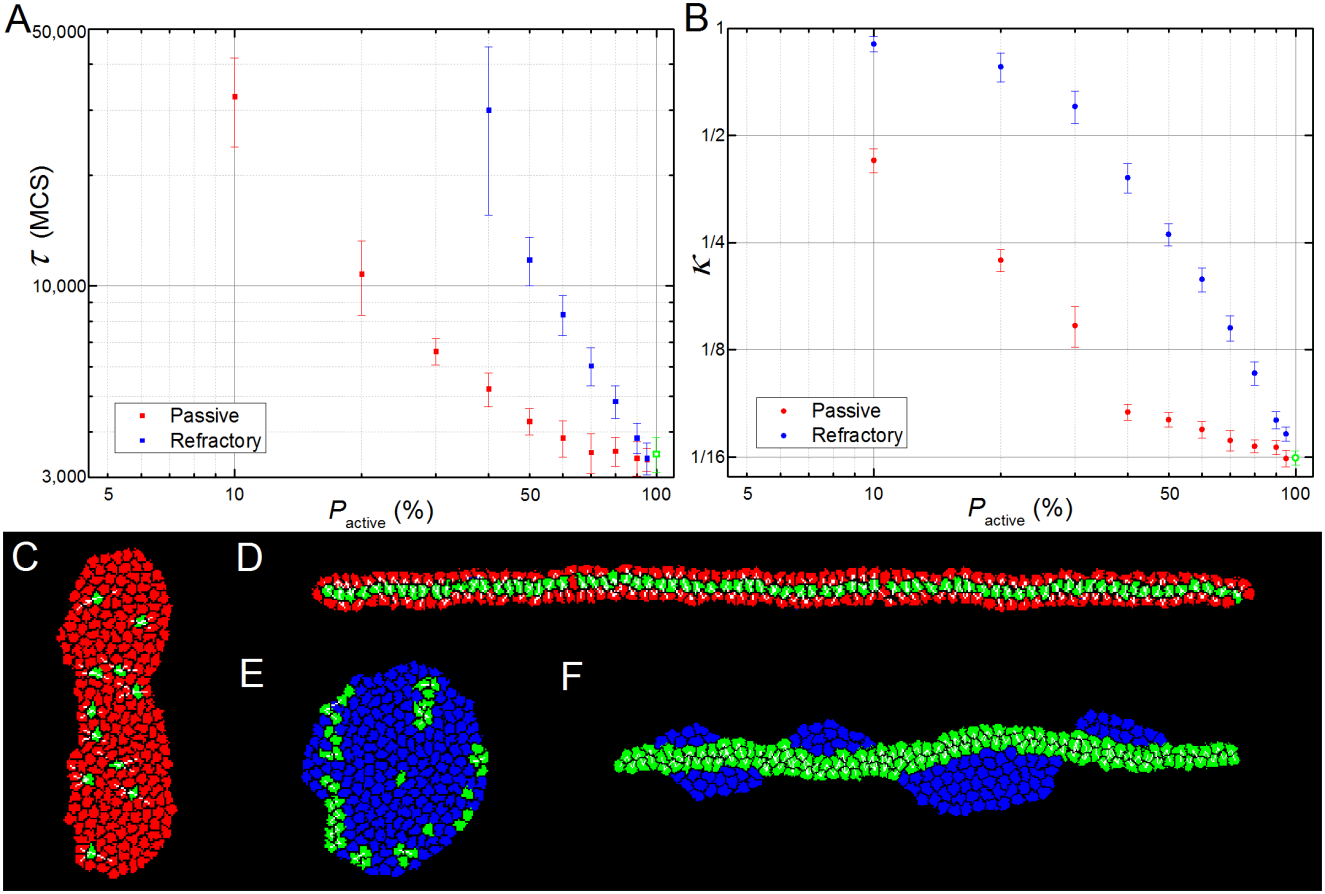
Simulation results for heterogeneous tissues. (**A**) Dependency of parameter κ with the percentage of passive (red dots) and refractory (blue squares) **cells**. (**B**) Dependency of parameter τ with the percentage of passive (red dots) and refractory (blue squares) **cells**. For both graphs, the measured value for the homogeneous **tissue** is represented by the green open square (**A**) or dot (**B**). Values of κ are measured for the whole **tissue** (active and non-active **cells**). (**C**-**D**) Simulations with passive **cells**; (**E**-**F**) simulations with refractory **cells**. (**C**) On a simulation with 95% of passive **cells** (in red) the remaining 5% of active **cells** (green) are still able to induce some degree of CE. (**D**) In a typical simulation with a higher percentage of active **cells** (here 33%) the active **cells** align at the center line of the extending **tissue**. (**E**) A failed CE for a tissue with less than 20% of active **cells** (here, 82% of refractory **cells**, blue). (**F**) When the percentage of active **cells** is above 20% (here 54%) the two populations sort out, with the active **cells** forming an elongated **tissue** and the refractory **cells** lying on each side of the structure. Panels (**D**) and (**F**) were rotated 90° for visualization purposes.

For **tissues** with a mixture of active and *passive* **cells**, both *κ* and *τ* decrease monotonically with the percentage of active **cells** in the **tissue** (Fig 9, red dots). However, even a fraction of active **cells** can drive CE. For 40% or more active **cells** (S2 Movie), the **tissue** deforms almost as much as a **tissue** composed entirely of active **cells** (Fig 9B, red dots), though the elongation time increases with the percentage of passive **cells** up to twice that for a **tissue** of all active **cells** (Fig 9A, red dots). For higher fractions of passive **cells** the final inverse aspect ratio increases significantly with the fraction of passive **cells** (Fig 9B). *E*.*g*., for 90% passive and 10% active **cells** (Fig 9C and S3 Movie), the **tissue’s** final inverse aspect ratio never drops below 0.3 (Fig 9B) and the elongation time *τ* is more than ten times that for a **tissue** of all active **cells** (Fig 9A). In all simulations, the active **cells** migrate towards the midline of the elongating **tissue**, leaving the passive **cells** at the lateral margins (Fig 9D).

The presence of relatively high CE despite the presence of only 10% of active **cells** can be explained by the relative **tissue** area covered by the **filopodia**. Every active **cell** can pull on neighbors that lie up to a distance *r*_max_ from its center of mass and within an angle *ϑ*_max_ on each side of its convergence plane (see Fig 2), thus covering an area of *2ϑ*_max_*r*_max_^2^. For the reference parameters (*ϑ*_max_ = *π*/2 and *r*_max_ = *2 cd*, see Table 1) and a population of 10% of active **cells** (*N/10*, where *N* is the number of **cells** in the **tissue**) this amounts to *~1*.*2N(cd)*^*2*^, which more than covers the whole area of the **tissue** (*N(cd)*^*2*^).

*Refractory* **cells** have a stronger effect on CE than passive **cells**. CE fails when the percentage of refractory **cells** is above 60% (Fig 9B, blue squares), while with passive **cells** it only fails for percentages higher than 90% (Fig 9B, red squares). For higher fractions of active **cells**, the two populations sort out, with the active **cells** extending normally and the refractory **cells** displaced to both sides of the elongating **tissue** (Fig 9F). Surface tension between the **cells** and the surrounding **medium** causes the refractory **cells** to form droplet-like clusters which bend the extending active-**cell tissue** into a wavy bar (Fig 9F and S4 Movie).

### 3D Versions

**Fig 10:**
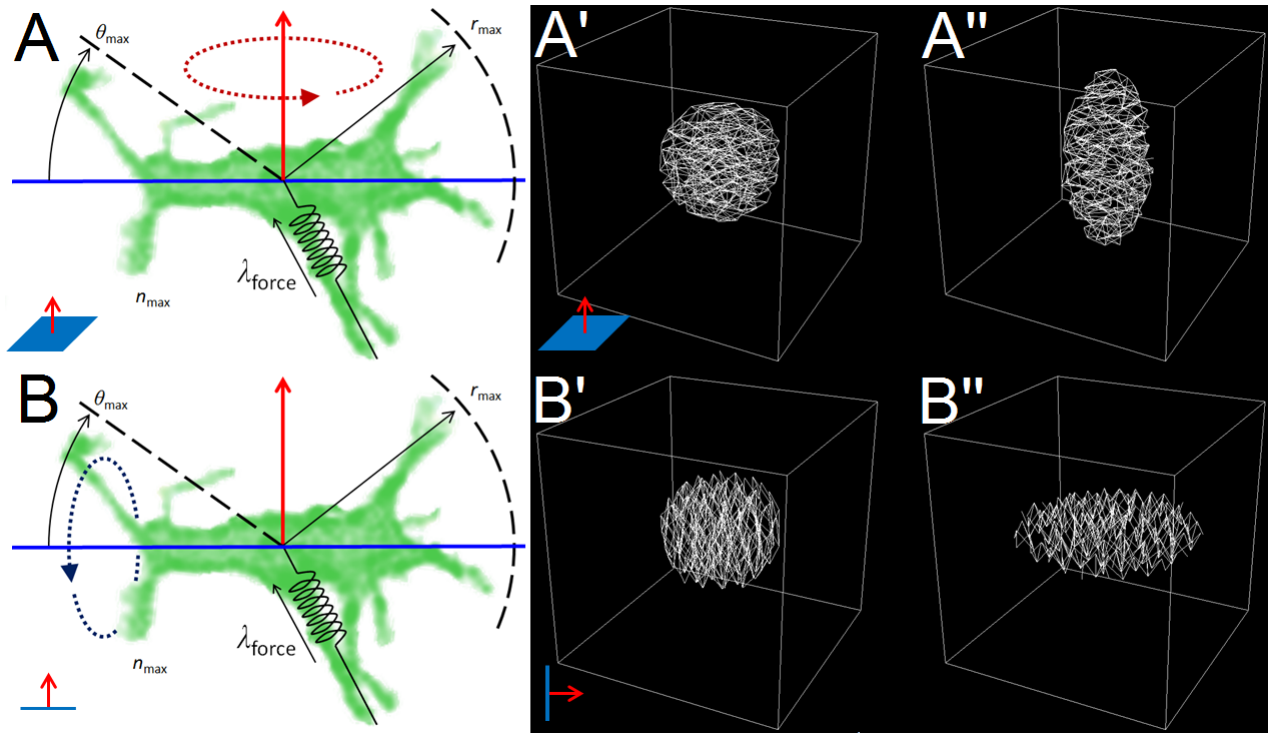
3D filopodial tension model versions. (**A**) Rotation around the polarization vector produces the 3D equatorial model. (**B**) Rotation around the convergence line results in the 3D bipolar model. (**A’-A”**) Initial and final states of a simulation of the equatorial model with all **cells’** polarization vectors pointing up. (**B’-B”**) Initial and final states of a simulation of the bipolar model with all **cells’** convergence axis lying vertically.

The 2D filopodial tension model is a reasonable description of cells within epithelial sheets, where cell movement is confined to a plane. However, in many situations cell intercalation occurs in 3D. That is the case in *radial intercalation* during epiboly of the developing *Xenopus Laevis* embryo, where cells in a multilayered epithelium intercalate and converge perpendicular to the plane of the sheet [43]. The filopodial tension model can be easily extended to three dimensions, but due to the extra degree of freedom, it breaks in two versions, depending on which axis is rotated:

In *equatorial* or *extensional* intercalation, obtained by rotating the 2D model around the polarization vector (the red arrow in Fig 2), the **cells** pull on all **neighbors** that lie in a *convergence plane* (Fig 10A). At the tissue level, equatorial intercalation results in the convergence of the **tissue** along the two directions perpendicular to the polarization vector and its extension along the polarization vector (Fig 10A’ and 10A”).

In *bipolar* or *convergent* intercalation, obtained by rotating the 2D model around the convergence line (the blue line in Fig 2), the **cells** pull on all **neighbors** that lie along a *convergence axis* (Fig 10B). At the tissue level, the bipolar intercalation results in the convergence of the **tissue** along the axis of convergence and its expansion in the other two directions (Fig 10B’ and 10B”).

Beginning with a spherical **tissue** with all the **cells** polarized in the same vertical direction, the 3D equatorial model produces a **tissue** resembling a prolate spheroid (cigar shaped, Fig 10A”, S5 Movie), while the bipolar model produces a **tissue** resembling an oblate spheroid (lentil shaped, Fig 10B”).

The bipolar model has more biological correspondence than the equatorial model: cells with unipolar or bipolar protrusive activity are much more common during development than cells with equatorial protrusive activity, and the resulting **tissue** shape from the 3D bipolar model corresponds to the *thinning and expansion* associated with radial intercalation.

**Fig 11:**
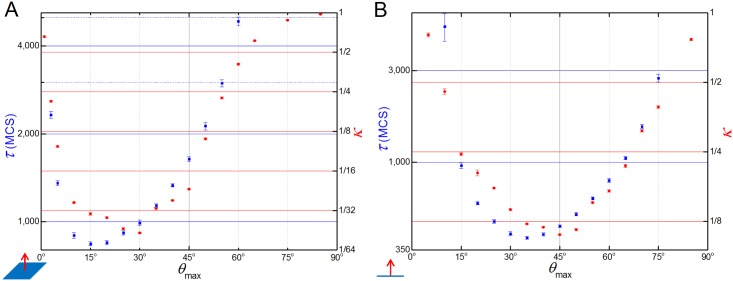
Dependence of τ and κ with ϑ_max_ in the 3D versions. (**A**) 3D extensional model and (**B**) 3D bipolar model dependence of κ and τ parameters with ϑ_max_. The range of best values for both κ and τ lies at much shorter angles in the 3D extension model (**A**) than the 2D model (see Fig 5D), which in turn has a range of optimal values slightly lower than in the 3D convergence model (**B**).

For both versions of the 3D model, the dependence of the parameters *κ* and *τ* with *λ*_force_, *r*_max_, *n*_max_ and *t*_interval_ are qualitatively the same as in the 2D model. The results only differ qualitatively with respect to *ϑ*_max_. For the same values of *r*_max_ and *n*_max_, the 3D convergence model is slightly less skewed than the 2D version, with the best value for *κ* around *ϑ*_max_ *=* 45° and the best value for *τ* around *ϑ*_max_ *=* 35° (Fig 11B). The 3D extension model, however, presents a more drastic change in the (*κ* and *τ*) vs. *ϑ*_max_ curve when compared to the 2D. While the 3D convergence model was slightly more symmetrical around *ϑ*_max_ *=* 90°, the 3D extension model is very skewed towards small angles, with the best values for *κ* around *ϑ*_max_ *=* 30° and the best value for *τ* around *ϑ*_max_ *=* 15° (Fig 11A). In the extensional model CE fails for *ϑ*_max_ *<* 3° and *ϑ*_max_ *>* 60°, while in the bipolar model CE fails for *ϑ*_max_ *<* 10° and *ϑ*_max_ *>* 75°.

The reasons for the asymmetry is that the final shape of the **tissue** in the 3D extension model—a two **cell** diameter tube orthogonal to the convergence plane—is more sensitive to perturbations than the lentil shape tissue obtained by the 3D bipolar model. Two **cells** pulling each other along a convergence axis leads them to be aligned in a plane perpendicular to the direction of the pulling. This plane fully coincides with the 3D bipolar model extension plane (Fig 10B”), but only partially with the extension plane of the 3D extension model (Fig 10A”). In the simulations results shown in Fig 11A, the value of ϑ_max_ = 30° represents the optimal maximum angle value where the pulling forces in the 3D extension model are still able to align the **tissue** without destabilizing it.

## Discussion

Here we developed a new 2D model of for active cell intercalation. The model drastically differs from previous existing models by its explicit use of pulling forces between cells rather than anisotropy on adhesion energies or surface tensions on the cell surface. A recent experimental work by Pfister *et al*. [28] supports our force-driven model. The use of forces makes the model easily adaptable to three dimensions, but the extra degree of freedom gives two ways in which this can be achieved: either by rotating the model around the polarization vector or around the convergence line (see Fig 10A and 10B).

The model was implemented in CompuCell3D using the Cellular Potts formalism. The core of the model, however, is independent of the mathematical formalism and can be easily implemented on other types of agent-based formalisms such as cell-center or vertex models, as long as they provide a volume exclusion mechanism. Although some quantitative results might differ, we expect the same qualitative results. The advantages of implementing and simulating our model using the Cellular Potts formalism include the ease to manipulate and study the effects of the surface tension on the tissue dynamics (Fig 4) and the addition of the model as an integrated part of the CompuCell3D simulation package [44] that allows for it to be immediately reusable by others.

Model validation can be done at two different levels quantitative and semi-quantitative: when both microscopic and macroscopic measurements are available for a specific tissue, using model parameters that agree with those measured for the cells in the tissue should result in tissue-level model output (here, the rate of convergence and final aspect ratio) agreeing quantitatively with that of the experimental tissue. This agreement should persist under different experimental conditions. This form of validation shows that the hypothesized mechanisms included in the model are sufficient to reproduce the experiment quantitatively. Note that a model can only show the sufficiency of modeled mechanisms, not their necessity, since a different set of mechanistic hypotheses might yield the same results. In our case, since we are not modelling a specific tissue, model validation can only be semi-quantitative. We show that changes in the properties of the modeled **cells** (such as average number of **filopodia**, length and angular distributions) and **tissue** properties (such ratio of active and non-active **cells**) predict relative changes in the rate of CE and final tissue aspect ratios in real tissues that agree with those in experiments when the corresponding parameter and conditions are similarly modified. In this case, agreement demonstrates the plausibility of the hypothesized mechanisms, but detailed quantitative validation requires additional experimental measurements. We hope that our semi-quantitative analysis of the filopodial tension model of CE will inspire the additional experimental measurements that a more detailed quantitative validation requires.

Our model predicts that external forces, such as surface tension and pressure, can only affect the final degree of tissue elongation (*κ*) (Fig 4), whereas the internal parameters that regulate cell-intercalation can affect both tissue dynamics (as measured by *τ*) and the final tissue shape (Figs 4 and 5). This can be easily tested experimentally by either changing the properties of the external environment (the surrounding cells/matrix) of the intercalating tissue or culturing it *ex vivo*.

Of the five **cell** intercalation parameters, the time interval between link formation/breakage (*t*_interval_) had negligible effects as long as it is below the typical time that the cells take to rearrange positions and/or shapes in response to a given set of external forces. This might be different if a refractory time interval between pulls is added to the model. We expect that in the presence of such refractory time, an increased frequency in link formation/breakage would slow down the speed of intercalation and reduce the final elongation ratio.

The model also predicts that the maximum range of cell interaction (*r*_max_) and the maximum number of links per cell (*n*_max_) had no effect on the final tissue elongation after *r*_max_ *=* 2 and *n*_max_ *=* 3, but the time of elongation kept decreasing for higher values of *r*_max_ and *n*_max_ (Fig 5B and 5C). It was not possible to increase those parameters indefinitely to check if the speed of intercalation would also saturate because the simulated cells start to fragment past *r*_max_ *≥* 6 or *n*_max_ *≥* 7. The current implementation of the model, however, does not allow for more than one active link between cell pairs, which would likely decrease elongation time.

All cell intercalation models assume some type of increased cell activity along the convergence axis, which is often translated, as is the case here, into the assumption that the cells are bipolar (one exception being our 3D equatorial/extensional filopodial tension model). This however is not necessarily true and we expect the model to also work in cases where the simulated cells are either monopolar in opposite directions of the same convergence axis or randomly alternate being monopolar in each direction of the convergence axis.

Our model also suggests that CE can be successfully achieved even in the presence of relatively high degrees of polarization defects. We predict that tissues containing polarization misalignments of up to ±10° will be practically indistinguishable to the optimally aligned case. Even severe misalignments (about ±50°) would still lead to some CE, although to a much lesser degree of final elongation ratio and with longer tissue elongation times (Fig 8). Experimental disruptions of the PCP pathway that alter the global alignment of cells in a dose-dependent manner would provide a way to test some of these predictions. Addition of a simple mechanical feedback mechanism by which the cells readjust their polarization in response to the pulling forces from the neighbors does not have major effects on the speed of tissue elongation (Fig 8C), but can fully rescue and even improve on the final elongation ratio of tissues with low or even severe polarization misalignments (Fig 8B). We choose to implement a minimal phenomenological feedback mechanism to explore the general response and self-organization of the tissue, but the formulation of the model allows the replacement of this generic mechanism with more detailed feedback models that reflect a specific tissue.

In cases where some cells fail to polarize, the severity of the effects on CE will depend on the type of interaction between the polarized (intercalating) cells and the unpolarized (non-intercalating cells). If the polarized (or active) cells can still pull on the unpolarized cells, then CE still happens even in a situation where the vast majority of cells (95%) are not active (Fig 9), although at a great reduction in both speed and final elongation ratio. If, on the other hand, the unpolarized cells are non-responsive and cannot be pulled, then the reductions in speed and final elongation ratio are much more sensitive to the presence of unpolarized cells (Fig 9) and CE completely fails when the population of active cells falls below 25% (Fig 9C). Another prediction of the model is the separation between the intercalating cells and the non-responsive/refractory cells (Fig 9D). Such defects could be induced experimentally by randomly distributed knock-out of intercalating cells, *e*.*g*. using electroporation of tissues with a dominant negative or RNAi, and would provide a way to further test model predictions.

Finally, the model reduces to the more common implementations when the maximum range of interaction is replaced by the common contact area condition. In this case, instead of contracting (or increasing the tension of) the cell’s surfaces that are aligned with the polarization vector or the global direction of convergence, we pull the neighbors that are not aligned with it (Fig 6D). In both cases active CE is achieved by the same principle, promoted cell-cell activity along one axis and inhibition along the other.

## Supporting Information

S1 Text (DOCX)

S1 Fig Independence of simulation results on random number generator seed. Four simulations with different seeds for random number generator produce similar results.
(TIF)

S2 Fig GGH/CP model. Each cell is an extended domain of sites on a *cell lattice* that share a common index, indicated one right panel by the numbers 1–6. Each cell, in turn, can be associated to a cell type, which are here displayed as different colors. Cells of the same type are set to have the same properties.
(TIF)

S3 Fig Size effects on the simulation results. (**A**) The parameter τ (blue open squares) increases exponentially, while the parameter κ (red dots) decreases exponentially with the number of cells. (**B**) A qualitative similar result for τ is obtained when the cell resolution (cell diameter, measured in lattice sites) is increased in the simulation. Left vertical axes and open blue squares correspond to τ values and right vertical axes and solid red dots corresponds to κ values.
(TIF)

S1 Movie Typical filopodial-tension model simulation. Left panel shows **cells** in green and **filopodial** links connecting **cells** center-of-mass in white lines. Right panel shows **cell** borders in cyan and **filopodial** links in white. Parameters as in Table 1. Time frames progress in logarithmic scale.
(MP4)

S2 Movie Simulation of CE of a tissue with 67% of passive cells. Active **cells** in green, passive **cells** in red and **filopodial** links in white. **Tissue** converge-extends almost as much as **tissues** composed of only active **cells**.
(MP4)

S3 Movie Simulation of CE of a tissue with 90% of passive cells. Active **cells** in green, passive **cells** in red and **filopodial** links in white. CE still happens even with a very low population of active **cells**.
(MP4)

S4 Movie Simulation of CE of a tissue with 50% of refractory cells. Active **cells** in green, refractory **cells** in blue and **filopodial** links in white. Active and passive **cells** sort out, with refractory **cells** forming droplet-like clusters which bend the extending active-**cell tissue** into a wavy bar.
(MP4)

S5 Movie 3D equatorial intercalation model. Left panel shows a cross-section of the tissue. Right panel shows a 3D view where only the filopodial links are visualized.
(MP4)
