# Supplementary material for: Filopodial-Tension Model of Convergent-Extension of Tissues
Source: PLoS Comput Biol. 2016 Jun 20;12(6):e1004952. doi: 10.1371/journal.pcbi.1004952 (PMC4913901; doi:10.1371/journal.pcbi.1004952)
Supplement: S1 Text — (DOCX) [file pcbi.1004952.s001.docx]

**Supplementary Text S1**

**Models of convergent-extension**

Our filopodial-tension model of convergent-extension (CE) is based on the experimentally-observed high protrusive and contractile activity of intercalating cells along the axis perpendicular to the axis of tissue elongation. Such protrusive activity is characteristic of deep mesenchymal cells and differs from the cell shape changes observed during CE of epithelial sheets, which do not display medio-lateral protrusions to the same extent. This difference suggests that different tissues may employ different cell-level mechanisms in order to drive CE: deep cells intercalate by actively pulling neighboring cells along a converging axis, thus extending the tissue in the direction perpendicular to it; while epithelial cells found in monolayers intercalate by contraction of cell surfaces parallel to the converging axis.

Most models of CE implement variations of this second mechanism, where cell edges or sides parallel to the converging axis shrink through a model-specific mechanism [1-4]. Models that differ significantly from this approach include the ones proposed by Vroomas *et al.* [5], based on increased cell adhesion in segmented tissues; and by Backes *et al.* [6], based on undirected cell elongation in the presence of a constraining boundary. Models based on the anisotropic cell shape of deep cells, include the ones proposed by Weliky *et al.* [7], which combine a constraining boundary with cells that protrude some of their vertices or leading edges, along a preferred direction; and by Honda *et al.* (mechanism II in [3]), where cells undergo repeated rounds of elongation along the converging axis followed by relaxation periods.

As far as we know, the only modeling paper that explicitly discusses the protrusive activity of intercalating cells is the one by Brodland [8]. While Brodland's model is inspired by the experimentally-observed presence of lamellipodia in intercalating cells, it is not a protrusion/contraction-driven model, as the implemented mechanism is the same as that in the cell intercalation models based on edge/surface contraction, *e.g.* those of Honda *et al.* (see mechanism I in [3]) and Rauzi *et al.* [4]. Brodland’s model employs the standard approach to simulation of edge contraction used in vertex models [9]: the “lamellipodial-pull” between two distant cells A and D, is modeled as the contraction of the edge between the two neighboring cells B and C, as shown in figure 1 of [8]. Therefore, while the work of Brodland is significant, its mechanism does not directly translate the experimentally-observed pulling forces between cells due to filopodia into forces in between cells in the model, but rather models cell-cell forces by the contraction of surfaces parallel to the converging axis of the tissue, similar to the approach taken in other vertex models [3,4,7]. While this approach may provide useful tissue-level results, the use of an averaged tension along a cell surface rather than an explicit representation of filopodia means that the model would be difficult to extend to situations where the explicit properties of the filopodia are important (*e.g.* when they bind to a spatially distributed set of ECM targets or when their angle, length and lifetime distributions are critical).

The present model, on the other hand, is inspired by experimentally-observed presence of filopodial protrusions in intercalating cells and directly translates this mechanism by modeling these protrusions as point-to-point tension forces between interacting cells. As such, our filopodial-tension model is fundamentally distinct from previous models of CE and generates novel predictions beyond those of edge contraction models. Unlike previous models, cell interactions are not necessarily limited to adjacent cells, but can extend beyond the immediate neighborhood of the cell and can interact with more than 2 cells at a time and between cells and spatially structured ECM. Our model can also easily simulate tissues in 3D, a feature that was only explored in the work by Honda *et al.* [3], which simulated in monolayers arranged in a 3D surface, a limited subset of the phenomena we explore.

Besides these differences in implementation, we studied extensively how tissue-level filopodial-tension model outcomes vary with parameters and environmental conditions, such as polarization misalignment, presence of non-active cells and external forces. Another feature that sets our work apart from previous models of CE is the addition and study of the effects of a simple feedback mechanism between intercalating cells. While none of those explorations can be found in previous models of CE, it would be interesting to know how edge contraction models respond to the same conditions.

**Glazier-Graner-Hogeweg (*GGH*) / Cellular Potts (CPM) Computational Model**

The GGH computational model represents space as a regular lattice of sites, or pixels (S2 Fig). A GGH *generalized cell* may represent a biological cell, a subcellular compartment, a cluster of cells, or a piece of non-cellular material or surrounding medium. Each generalized cell is an extended domain of sites on a *cell lattice* that share a common index (referred to as the *cell index*, *σ*). The cell-lattice configuration corresponds to an *effective energy* (*H*), defined so that simulated **cells** have the desired properties, behaviors and interactions, implemented via constraint terms in *H*. The effective energy in GGH simulations is not the actual energy of the biological cells and tissue being modeled but a simple way to specify the factors that govern **cell** properties, behaviors and dynamics in the simulated biological model. In our model **cells** have volumes, and interact via adhesion and dynamical cell-cell pulling forces, so that *H* is given by the following equation:

$H=\sum_{\begin{matrix} \vec{i},\vec{j} \\ \text{neighbors} \end{matrix}} J\left( \tau\left( \sigma_{\vec{i}} \right),\tau\left( \sigma_{\vec{j}} \right) \right)+\sum_{\sigma} \lambda_{\text{vol}}(\sigma)\left( v\left( \sigma\right)-V_{\text{t}}(\sigma) \right)+\sum_{\sigma,\sigma'} \lambda_{\text{force}}(\sigma,\sigma')l_{\sigma,\sigma'}$ (Equation S1)

The first sum, over all pairs of neighboring lattice sites and $\vec{j}$ $\vec{j}$, calculates the *boundary* or *contact* *energy* between neighboring **cells** $\sigma\left( \vec{i} \right)$ $\sigma\left( \vec{i} \right)$. $J\left( \tau\left( \sigma_{\vec{i}} \right),\tau\left( \sigma_{\vec{j}} \right) \right)$ $J\left( \sigma\left( \vec{i} \right),\sigma\left( \vec{j} \right) \right)$ is the boundary energy per unit contact area for **cells** of types $\tau\left( \sigma_{\vec{i}} \right)$ $\sigma\left( \vec{i} \right)$ and $\sigma\left( \vec{j} \right)$ $\tau\left( \sigma_{\vec{j}} \right)$ occupying sites $\vec{i}$ $\vec{i}$ and $\vec{j}$ $\vec{j}$, respectively$\sigma\left( \vec{j} \right)$, and is only evaluated for lattice sites belonging to different **cells** (sites belonging to the same generalized **cell** are assumed to have zero contact energy). We specify $J\left( \sigma\left( \vec{i} \right),\sigma\left( \vec{j} \right) \right)$ $J\left( \tau\left( \sigma_{\vec{i}} \right),\tau\left( \sigma_{\vec{j}} \right) \right)$ as a matrix indexed by the **cell** **types**. Higher (more positive) contact energies between **cells** result in greater repulsion between the **cells** and lower (more negative) contact energies between **cells** result in greater adhesion between the **cells**.

The second sum in (Eq. S1), over all **cells**, calculates the effective energies due to the volume constraint. Deviations of the volume of **cell** σ from its target value *V*_t_*(σ)* $\text{V}_{\text{t}}\left( \text{σ} \right)$ $\text{V}_{\text{t}}\left( \text{σ} \right)$increase the effective energy, penalizing these deviations. On average, a **cell** will occupy a number of pixels in the cell lattice slightly smaller than its target volume due to surface tensions from the contact energies (*J*). The parameter *λ*_vol_ $\text{λ}_{\text{vol}}$behave like Young’s moduli, with higher values reducing fluctuations of a **cell**’s volume about its target value. The third sum in (Eq. S1) over all connected **cells**’ pairs represent the pulling forces between intercalating **cells** and will be described in more detail later.

**Cell** dynamics in the GGH model provide a much simplified representation of cytoskeletally-driven cell motility using a stochastic modified Metropolis algorithm consisting of a series of index-copy attempts. Before each attempt, the algorithm randomly selects a target site, $\vec{i}$ $\vec{i}$, and a neighboring source site $\vec{i}$ $\vec{i}$. If different **cells** occupy those sites the algorithm sets $\sigma\left( \vec{i}' \right)=\sigma\left( \vec{i} \right)$ $\sigma\left( \vec{i}' \right)=\sigma\left( \vec{i} \right)$ with probability, given by the Boltzmann acceptance function:

$P\left( \sigma\left( \vec{i} \right)\to\sigma\left( \vec{i}' \right) \right)=\left\{ \begin{aligned} e^{-\frac{\Delta H}{T_{m}}} : \Delta H>0 \\ 1 : \Delta H\leq0 \end{aligned} \right.$ , (Equation S2)

where *ΔH* is the change in the effective energy if the copy occurs and *T* is a global parameter describing **cell** membrane fluctuations that we will discuss momentarily. A Monte Carlo Step (*MCS*) is defined as *N* index-copy attempts, where *N* is the number of sites in the cell lattice, and sets the natural unit of time in the computational model.

The Metropolis algorithm evolves the cell-lattice configuration to simultaneously satisfy the constraints, to the extent to which they are compatible, with perfect damping (*i.e.*, average velocities are proportional to applied forces).

A potential index copy that increases the effective energy, *e.g.*, by increasing deviations from target values for **cell** volume or surface area or juxtaposing mutually repulsive **cells**, is improbable. Thus, the pattern evolves in a manner consistent with the biologically-relevant “guidelines” incorporated in the effective energy: **cells** maintain surface areas and volumes close to their target values, mutually adhesive **cells** (with low **cell**-**cell** contact energy) stick together, mutually repulsive **cells** separate, *etc*..*.* Thus, the average time-evolution of the cell lattice corresponds to that achievable deterministically using finite-element or center-model methodologies with perfect damping.

For a further introduction to GGH modeling, see [10,11].

**Size Effects on CE**

The number of **cells** in the **tissue**, *N*, and the resolution of the model (the **cell** diameter, *cd*, in lattice sites), used in the CP/GGH representation of the **cell**) can also affect *κ* and *τ* (S3 Fig). The number of **cells** in the deforming **tissue** limits the **tissue’s** minimum final inverse aspect ratio to the value when all **cells** are stacked in a single column. Thus the minimum possible value of *κ* scales with *N* ^-1^, close to the measured slope of -0.9±0.01 from S3A Fig. The time the **tissue** takes to elongate (*τ*), however increases as a power law (*τ* $\propto$ *N* ^0.75±0.03^), because in a larger **tissue** more **cells** must displace a greater distance in order to achieve the same degree of **tissue** elongation.

A power law for *τ* is also observed with respect to the **cell** diameter (*τ* $\propto$ *cd* ^2.34±0.04^), as the drag scales as the area of the **cell** (S3B Fig, blue open squares). The **tissue’s** final deformation ratio (*κ*), however, remains relatively unchanged, since the final inverse aspect ratio should correspond to an energy minimum, which depends only on the balance between the **link** tension forces and surface tension, both of which are independent of the **cell** diameter (S3B Fig, red dots).

1. Zajac M, Jones GL, Glazier JA (2000) Model of convergent extension in animal morphogenesis. Physical Review Letters 85: 2022-2025.

2. Zajac M, Jones GL, Glazier JA (2003) Simulating convergent extension by way of anisotropic differential adhesion. Journal of Theoretical Biology 222: 247-259.

3. Honda H, Nagai T, Tanemura M (2008) Two different mechanisms of planar cell intercalation leading to tissue elongation. Developmental Dynamics 237: 1826-1836.

4. Rauzi M, Verant P, Lecuit T, Lenne PF (2008) Nature and anisotropy of cortical forces orienting Drosophila tissue morphogenesis. Nat Cell Biol 10: 1401-1410.

5. Vroomans RM, Hogeweg P, Ten Tusscher KH (2015) Segment-specific adhesion as a driver of convergent extension. PLoS Comput Biol 11: e1004092.

6. Backes TM, Latterman R, Small SA, Mattis S, Pauley G, et al. (2009) Convergent extension by intercalation without mediolaterally fixed cell motion. Journal of Theoretical Biology 256: 180-186.

7. Weliky M, Minsuk S, Keller R, Oster G (1991) Notochord Morphogenesis in Xenopus-Laevis - Simulation of Cell Behavior Underlying Tissue Convergence and Extension. Development 113: 1231-&.

8. Brodland GW (2006) Do lamellipodia have the mechanical capacity to drive convergent extension? Int J Dev Biol 50: 151-155.

9. Fletcher AG, Osterfield M, Baker RE, Shvartsman SY (2014) Vertex models of epithelial morphogenesis. Biophys J 106: 2291-2304.

10. Swat MH, Hester SD, Balter AI, Heiland RW, Zaitlen BL, et al. (2009) Multicell simulations of development and disease using the CompuCell3D simulation environment. Methods Mol Biol 500: 361-428.

11. Swat MH, Thomas GL, Belmonte JM, Shirinifard A, Hmeljak D, et al. (2012) Multi-scale modeling of tissues using CompuCell3D. Methods Cell Biol 110: 325-366.
