# Supplementary figures and images for: Filopodial-Tension Model of Convergent-Extension of Tissues
Source: PLoS Comput Biol. 2016 Jun 20;12(6):e1004952. doi: 10.1371/journal.pcbi.1004952 (PMC4913901; doi:10.1371/journal.pcbi.1004952)

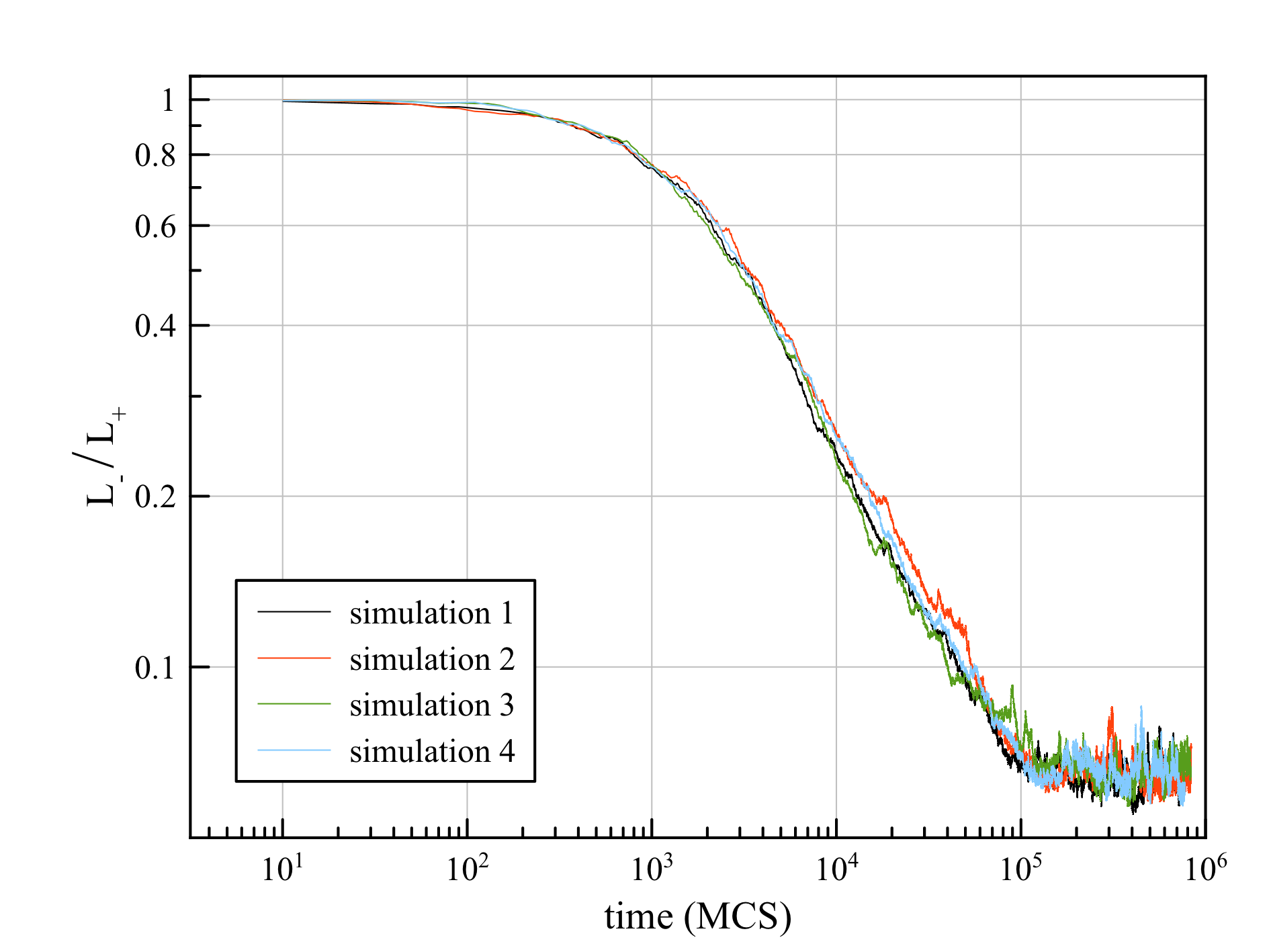

Supplement: S1 Fig — Four simulations with different seeds for random number generator produce similar results. (TIF) [file pcbi.1004952.s002.tif]

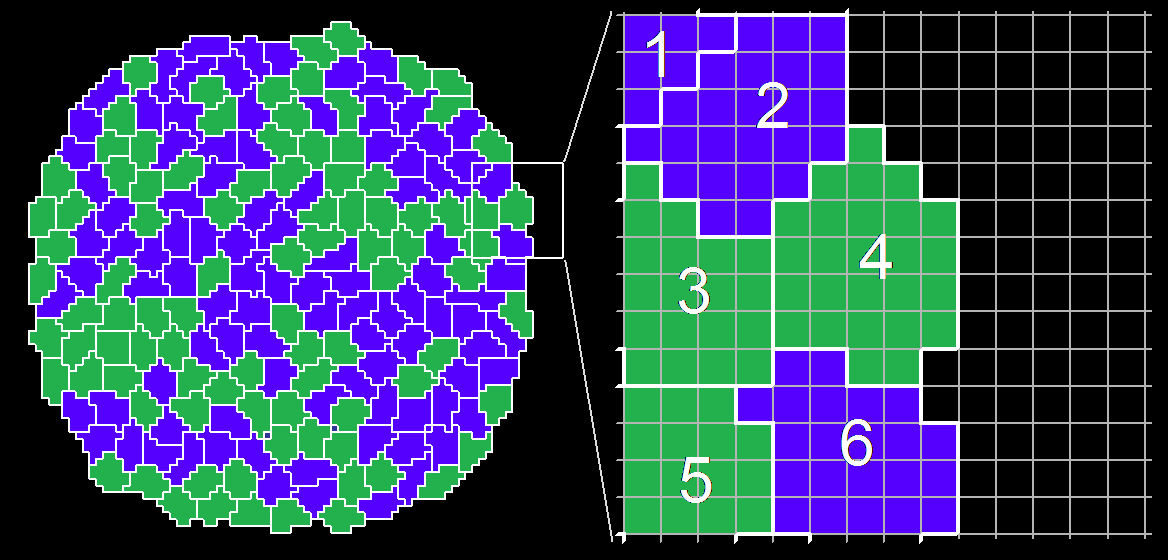

Supplement: S2 Fig — Each cell is an extended domain of sites on a cell lattice that share a common index, indicated one right panel by the numbers 1–6. Each cell, in turn, can be associated to a cell type, which are here displayed as different colors. Cells of the same type are set to have the same properties. (TIF) [file pcbi.1004952.s003.tif]

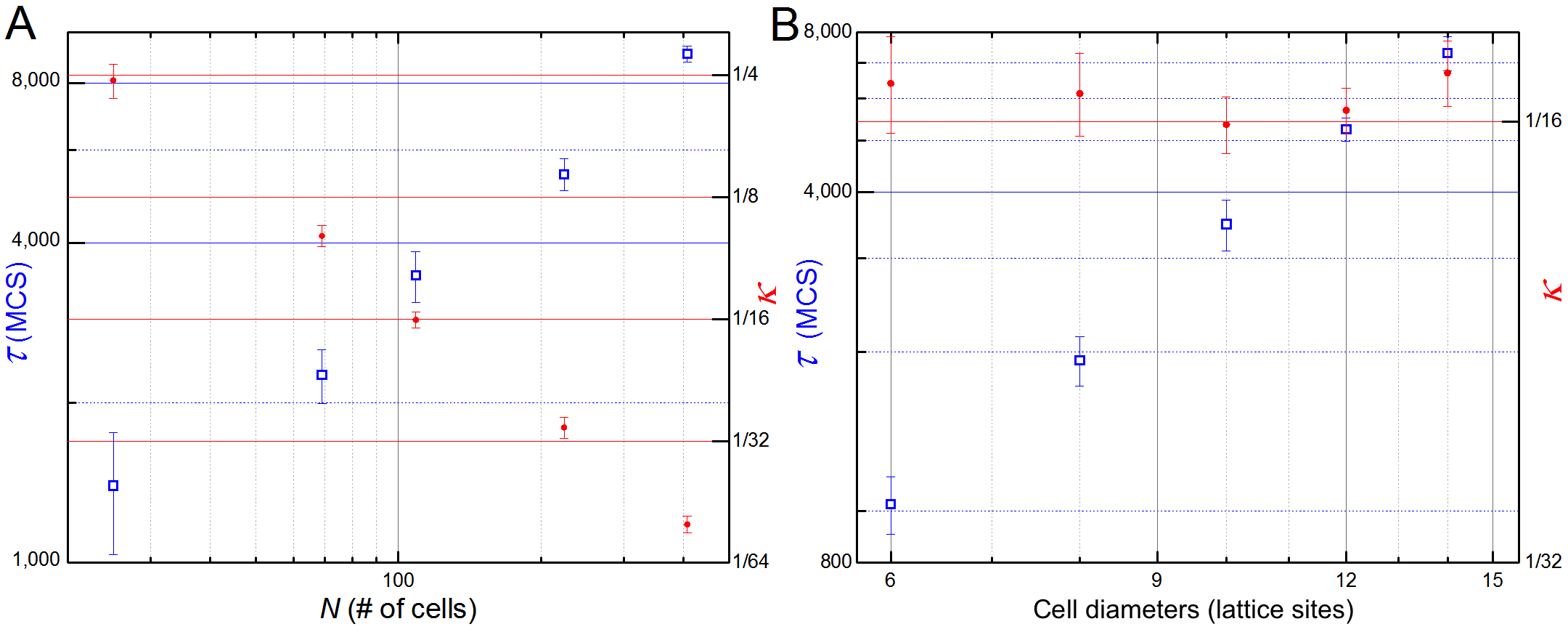

Supplement: S3 Fig — (A) The parameter τ (blue open squares) increases exponentially, while the parameter κ (red dots) decreases exponentially with the number of cells. (B) A qualitative similar result for τ is obtained when the cell resolution (cell diameter, measured in lattice sites) is increased in the simulation. Left vertical axes and open blue squares correspond to τ values and right vertical axes and solid red dots corresponds to κ values. (TIF) [file pcbi.1004952.s004.tif]
